# Experience with standardized assessments in child and adolescent psychiatry: Findings from a national trainee survey in Switzerland

**DOI:** 10.1007/s00787-026-03052-0

**Published:** 2026-06-03

**Authors:** Matthias Liebrand, Maya Cosentino, Michael Kaess, Stephan Eliez, CAP Residents Consortium, Denise Aiello, Marco Armando, Raphaël Béné, Audraine Le Boudec, Ana Coman, Luisa von den Driesch, Constance de Metz, Javier Gimenez, Seniye Kamek, Achudhan Karunaharamoorthy, Angelos Katsarakis, Alina Killer, Dominika Kresakova, Konstantinos Paschalidis, Louis Popescu, Ösnur Sönmez, Sophie Touzin-Andrews, Ašara Valudskytė, Paul Klauser, Jochen Kindler

**Affiliations:** 1 University Hospital of Child and Adolescent Psychiatry and Psychotherapy, University of Bern, Bern, Switzerland; 2 Department of Child and Adolescent Psychiatry, Centre for Psychosocial Medicine, University Hospital Heidelberg, Heidelberg, Germany; 3 Department of Psychiatry, Faculty of Medicine, Fondation Pôle Autisme, University of Geneva, Geneva, Switzerland; 4 Department of Psychiatry, Division of Child and Adolescent Psychiatry, Lausanne University Hospital and University of Lausanne, Lausanne, Switzerland; 5 Department of Psychiatry, Center for Psychiatric Neuroscience, Lausanne University Hospital and University of Lausanne, Lausanne, Switzerland; 6 Psychiatry Baselland, Hospital of Child and Adolescent Psychiatry, Liestal, Basel-Landschaft Switzerland

**Keywords:** Child and adolescent psychiatry, Standardized assessments, Training, Trainees, Residents, Assistant psychologists

## Abstract

The current shortage of child and adolescent mental health specialists in Europe and around the world, coupled with an increasing mental health burden among young people poses significant challenges that future child and adolescent psychiatry (CAP) training programs must address. Promoting the use of standardized assessments (SA) has the potential to improve both efficiency and quality of patient care as well as resident training programs. This study aimed to characterize a sample of CAP trainees (residents and assistant psychologists) in Switzerland and evaluate their SA use as well as their corresponding skills and attitudes. The project was launched at the Swiss CAP Association Residents’ Day 2022 in Zurich, where the development of a national survey was initiated. The survey was subsequently refined and distributed to all CAP training facilities in Switzerland. The final sample included 73 residents and 121 assistant psychologists. The majority of trainees from both groups reported being interested in using SA and convinced of their clinical usefulness. Common reasons for using SA in clinical practice were to support objectivity, structure clinical interviews, and address sensitive topics with patients. Participants’ SA use varied by type of psychotherapy training but did not differ by type of institution (university vs. non-university hospital) or academic degree. Self-rated ability to administer SA correlated positively with SA training opportunities. A frequently reported use barrier was the lack of SA translations. Based on our findings, CAP would benefit from teaching and promoting SA as a seminal, evidence-based practice in clinical and training program contexts.

**Supplementary Information:**

The online version contains supplementary material available at https://doi.org/10.1007/s00787-026-03052-0.

## Introduction

Given the steady decline of youth mental health in many countries in Europe and across the world [1], the need for ample, well-trained mental health professionals who can accurately assess and effectively treat young people suffering from mental illness is greater than ever. However, many countries are experiencing an increasing shortage of child and adolescent specialists [2–4], making strategies to improve and increase the attractiveness of child and adolescent psychiatry (CAP) training programs crucial. In Switzerland, there is a lack of child and adolescent psychiatrists, resulting in children and adolescents having to wait more frequently and longer for psychiatric assessment and/or treatment than adults [5]. Public surveys indicate that only 43% of CAP institutions in Switzerland can fill or mostly fill their resident positions with qualified candidates [5] and a certified child and adolescent psychiatrist shortage of up to 33% is expected in 2030 [2]. A similar scarcity can be found in the United States [3, 6] and across Europe [4, 7, 8]. Acknowledging the global scarcity of medical specialists in CAP, numerous expert representatives of international associations and organizations, including the World Psychiatry Association (WPA) and the World Health Organization (WHO), have defined the need to increase the CAP workforce as one of their top priorities [9].

Given these predictions and the increasing health care service demand, CAP is in need of well-trained psychiatrists. Alongside comparable financial compensation [10], CAP would benefit from investing in training programs and developing evidence-based teaching and practice strategies [11] central to improving the field and increasing its legitimacy and attractivity.

Acknowledging the existence of underdiagnosis, misdiagnosis, and insufficient treatment within psychiatry, improvements to psychiatric care are needed [12]. One element of evidence-based mental health care is the development, validation, and use of standardized assessments (SA) [13–15]. SA are tools designed to assess psychopathology and neuropsychological functioning that can support diagnostic processes and help plan and monitor treatment [16, 17]. They always include standardized instructions, scoring, and interpretation and are developed to have satisfactory psychometric properties (i.e., reliability, validity, change sensitivity) [18, 19]. Examples of SA include (semi)-structured interviews, questionnaires, cognitive tests, and standardized observational assessments.

SA can be used to objectify, monitor, and increase the efficiency and quality of patient care [20, 21]. A powerful way to implement SA is measurement-based care (MBC), where SA are used as a direct means to interact with patients via systematic routine treatment progress and outcome monitoring [22]. MBC can guide treatment and has been found to significantly improve mental health treatment outcomes in both adult and youth settings [23–25].

Other advantages of using SA in CAP include their potential to increase diagnostic objectivity, reliability, and accuracy, as well as their applicability across age groups and developmental stages [16]. Moreover, SA can measure changes over time and thereby monitor development and treatment efficacy. SA can also be useful for communication with trainees, researchers, as well as patients and their families. Finally, SA can provide reliable, high-quality data sets that can be used and shared for research purposes, enabling large-scale projects between institutions, critical for gaining knowledge and facilitating communication in CAP. SA thus have wide-ranging potential to enhance patient care, clinical practice, and research, instrumental to the future development of CAP.

In line with the study focus on trainees, SA may be a key component for strengthening CAP training programs. Through undergoing systematic training in SA, trainees can improve their evidence-based thinking and clinical reasoning, and also reduce their susceptibility to diagnostic bias [26]. In addition, incorporating repeated SA measurements throughout treatment may promote critical thinking and reflective practice [27]. For example, psychology trainees report that using SA to monitor treatment progress helps them identify changes over time and supports discussions around unexpected progress or challenges with patients [24].

Despite generally favorable attitudes among mental health clinicians (especially with cognitive-behavioral therapy (CBT) backgrounds), routine use of SA remains uncommon [15, 17, 22, 28]. Typical reasons for not using SA for routine outcome monitoring include the lack of training and supervision as well as organizational barriers and inadequate resources (e.g., available translations of SA and license fees) [17, 23, 29]. Emphasizing SA in the context of evidence-based training and clinical supervision has been found to quickly increase positive SA attitudes and more gradually increase SA use and skills, however continued means are likely necessary to avoid use reduction over time [30]. Given the advantages of using SA in clinical mental health settings for youth, it is recommended to offer clinicians comprehensive SA training opportunities [23].

In summary, SA not only have the potential to significantly contribute to the access and delivery of high-quality and effective mental health care during times of increasing mental health staff shortages, but also to strengthen CAP training programs and the proficiencies of trainees. To further our understanding of SA in CAP, particularly among trainees, we conducted an online survey to characterize SA use as well as corresponding skills and attitudes among CAP trainees in Switzerland. We expected to find differences between trainees at university and non-university institutions and predicted that SA use and skills would be related to academic degree and research attitudes. We also hypothesized that SA training opportunities would correlate positively with self-rated ability to administer SA and that perceived clinical benefits of SA would be associated with using SA more frequently.

## Methods

### Survey and sample

This project was initiated at the Swiss Association for Child and Adolescent Psychiatry and Psychotherapy Residents’ Day in September 2022. CAP residents (RES) from all over Switzerland who attended the Residents’ Day developed the first draft of a survey to evaluate SA within CAP (see also Kindler et al. [31]). The survey was further developed, entered into REDCap, and subsequently distributed (via email) to RES and assistant psychologists (AP) working in Swiss CAP training institutions in April 2024. To ensure that the survey was delivered to all the RES and AP at each institution, individual contacts (either a RES or an executive person) were established at each of the 33 training institutions (see supplementary material for a list of the institutions). These individual contacts distributed the survey per email to the RES and AP in their respective institutions and reported that they had done so via a confirmation email in which the date and number of emails that had been sent out to RES and AP was specified. This process ensured full anonymity of the participants. Participants were originally informed that they had six weeks to fill out the survey but received a notification email that the deadline had been extended for an additional two weeks to increase participation. No compensation was provided for completing the survey.

The survey included 40 mandatory questions, including questions with one response option, multiple response options, a visual analogue scale (VAS), and free text questions (see supplementary material for the full survey). Eleven questions were conditional, i.e., appeared only when a specific answer was selected in the previous question. Survey completion time was around 10—15 min.

The survey was sent out to approximately 270 RES and 550 AP (numbers are not exact given 3 institutions did not specify how many RES and AP they sent the survey to and because the survey was sent to a small number of individuals who were not CAP RES or AP). Importantly, the Swiss Institute for Medical Training and Further Education (SIWF) lists that in 2023 293 CAP RES were in training [32], indicating that nearly all CAP RES received the survey. A total of 288 subjects participated in the survey, 58 of whom were excluded because they did not complete the whole questionnaire. Another 36 participants were excluded because they did not classify themselves as RES or AP (*n* = 34) or did not work in CAP (*n* = 2). The final sample included 121 AP and 73 RES. Thus, the approximate response rate was 22% for AP and 27% for RES.

### Data curation and statistical analysis

Implausible responses pertaining to subjects’ workload were rounded to the nearest plausible value (e.g., 98% to 100%). Statistical comparisons were conducted with either independent sample t-tests or factorial analyses of variance (ANOVA) in R. The ANOVAs included profession (AP, RES) as a between-subject factor and one of several other between-subject factors (e.g., psychotherapeutic training type; see specifications below). Post-hoc tests were calculated with the emmeans package [33] in R and corrected for multiple comparisons with the Bonferroni-Holm method. Correlations were computed using Kendall’s rank correlation in R, given data were non-normally distributed. The significance threshold was set at *p* < 0.05.

## Results

### Sociodemographic results

Sociodemographic data are summarized in Table 1. Trainees were mostly female (RES: 76.7%, AP: 82.6%). Workload (full time equivalent) was slightly higher among RES than AP (t_192_ = 2.1, *p* = 0.035). Nearly all AP obtained their professional degree in Switzerland (95%), whereas this only applied to a minority of the RES (37%) (t_192_ = 11.4, *p* < 0.001). RES who obtained their professional degree outside of Switzerland reported that they had studied in one of twenty countries, with Germany (20.5%), Romania (11%), and Greece (6.8%) being the most frequently reported. Participants’ institutions were located in 18 of the 26 Swiss cantons (RES: 16, AP: 18), with some institutions serving multiple cantons.

**Table 1:** Overview of sociodemographic data of residents and assistant psychologists

Characteristic	Residents (*n* = 73)	Assistant Psychologists (*n* = 121)
Gender (%)	76.7 ♀, 23.3 ♂, 0.0 ⚤	82.6 ♀, 15.7 ♂, 0.8 ⚤
Age (Mean, SD, range) in years	32.6 ± 6.5 (25—55)	29.9 ± 4.9 (25—57)
Degree in Switzerland (%)	37.0	95.0
Mean workload in FTE (Mean, SD, range) in %	87.0 ± 14.9 (40—100)	82.6 ± 13.3 (40—100)
Children (Mean, SD, range)	0.52 ± 0.97 (0—4)	0.17 ± 0.51 (0—2)

*FTE* full-time equivalent, *SD* standard deviation.

### Psychotherapeutic approach and workplace language

The most common type of psychotherapeutic training was a combination of different psychotherapeutic approaches (CT—e.g., CBT and systemic therapy (ST)) (RES: 35.6%, AP 24.8%), followed by CBT (RES: 19.2%, AP 25.6%) and psychodynamic psychotherapy (PP) (RES: 20.5%, AP 20.7%). The most predominant psychotherapeutic approaches at the workplace were CBT (RES: 32.9%, AP 31.4%) and PP (RES: 31.5%, AP 28.9%). The most spoken workplace language was German (RES: 60.3%, AP 62.8%), followed by French (RES: 32.9%, AP 27.3%), a combination of Swiss languages (RES: 4.1%, AP 4.1%), and Italian (RES: 2.7%, AP 5.8%).

### Research

Generally, research was reported to be conducted at over half of the institutions (RES: 60%, AP: 55%). However, research involvement was reported to be minimal (RES: 9.3/100 [SD 20.4, range 0—100], AP: 4.6/100 [SD 13.5, range 0—100], VAS from “not at all” (0) to “very much” (100)) and limited to only a few hours per week (RES: 3.2 h/week [SD 9.9, range 0—50], AP: 1.1 h/week [SD 3.6, range 0—25]). Close to 40% of the sample reported having no research experience (AP: 41.3%, RES: 39.7%), and while 30.1% of RES reported having obtained a doctoral thesis (Dr. med.), only 3.3% of AP reported having obtained a PhD.

### Standardized Assessments (SA)

**Fig. 1:**
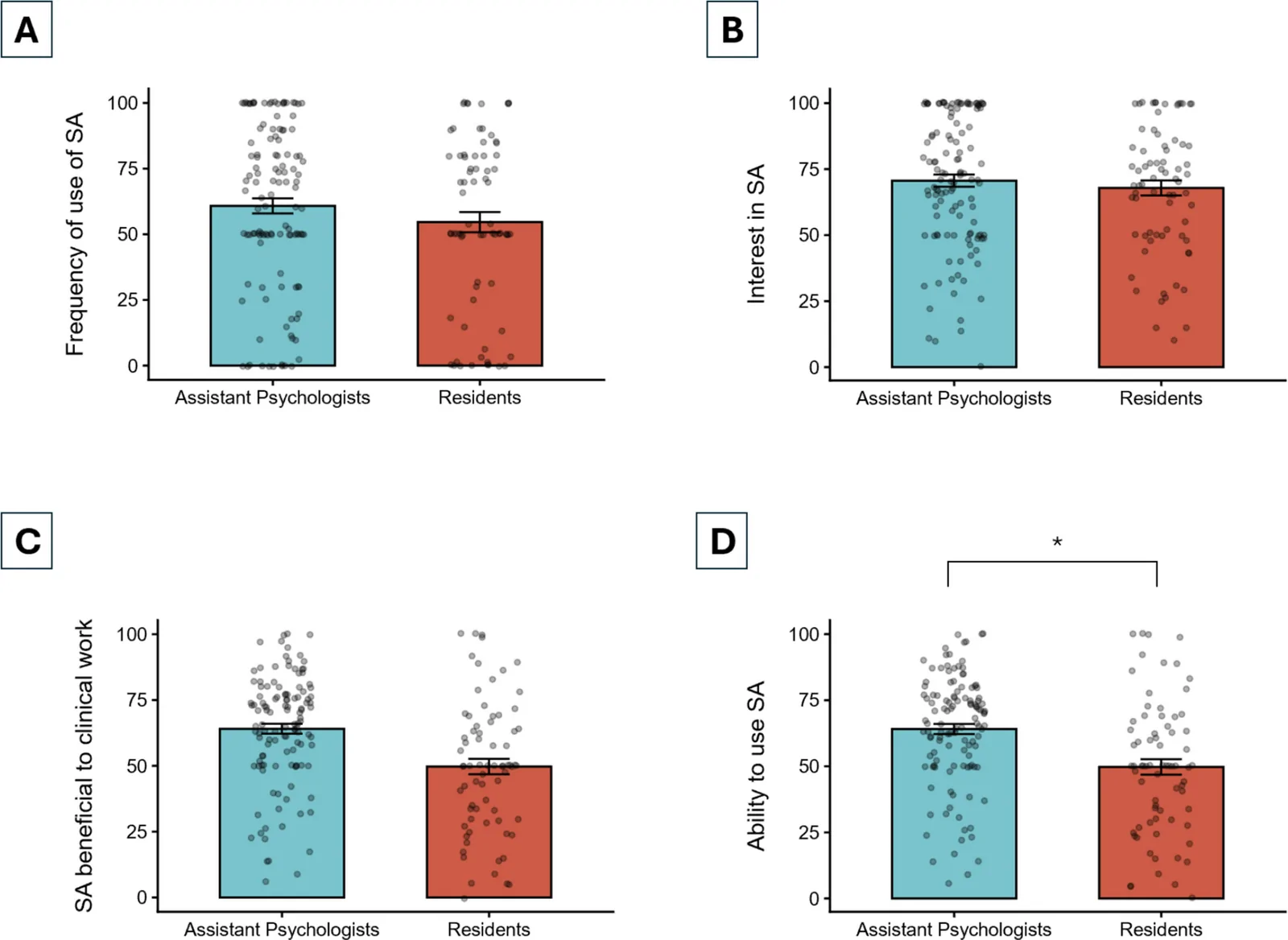
Barplots of frequency of use of SA (**A**), interest in SA (**B**), clinical usefulness of SA (**C**), and ability to use SA (**D**) among assistant psychologists and residents. The groups differed in their self-rated ability to use SA (**D**). Error bars depict the standard error of the mean. SA = standardized assessments

Trainees reported using SA fairly regularly (RES: 54.6/100 [SD 32.9, range 0—100], AP: 60.8/100 [SD 31.6 range 0—100], VAS from “never” (0) to “always” (100), see Fig. 1A). Frequency of use differed by type of psychotherapeutic training (F_4,756_ = 13.0, *p* < 0.001). Those with CBT training reported higher use than those in PP training (t_83_ = 5.9, *p* < 0.001), ST training (t_67_ = 3.5, *p* = 0.005), and unspecified psychotherapy trainings (UT, a residual category for participants who could not assign themselves to one of our specified training categories) (t_67_ = 4.9, *p* < 0.001). SA use was also higher among those in CT programs of several distinct schools compared to those in PP programs (t_94_ = 4.5, *p* < 0.001) and UT programs (t_78_ = 3.7, *p* = 0.005). Use also differed by the main psychotherapeutic approach at the institution (F_4,756_ = 4.2, *p* = 0.003), with higher use in workplaces with a predominantly CBT approach compared to a predominantly PP approach (t_118_ = 4.0, *p* = 0.001). Interestingly, there were no differences in SA use found between participants who worked in a university hospital and those who did not (F_1,189_ = 0.0, *p* = 0.88), or between participants with and without a scientific degree (Dr. med., PhD) (F_1,189_ = 0.9, *p* = 0.33). Finally, frequency of use differed by the workplace language (F_3,570_ = 8.1, *p* < 0.001), and was significantly higher in German speaking institutions than in French speaking institutions (F_1,189_ = 20.7, *p* < 0.001; this effect increased slightly when controlling for psychotherapy background: F_1,189_ = 24.2, *p* < 0.001).

Trainees reported personally administering SA in their clinical practice on a regular basis (RES: 52.5/100 [SD 30.9, range 0—100], AP: 60.7/100 [SD 30.7, range 0—100]) and regularly using the results of SA collected by others (RES: 41.6/100 [SD 31.1, range 0—100], AP: 53.4/100 [SD 30.7, range 0—100], all VAS from “never” (0) to “always” (100)). SA were reported to be commonly used at admission (RES: 74.0%, AP: 75.2%) and during treatment (RES: 79.5%, AP: 79.3%), but less so at discharge (RES: 24.7%, AP: 17.4%). SA were used mostly for diagnosis (RES: 84.9%, AP: 95.0%), screening (RES: 68.5%, AP: 80.2%), and psychotherapy treatment monitoring (RES: 32.9%, AP: 50.4%). Few subjects reported not using SA at all (RES: 8.2%, AP: 2.5%). The different types of SA that RES and AP reported using in clinical practice are displayed in Table 2 (note that general types and not specific instruments were assessed).

**Table 2:** Overview of usage of different types of standardized assessments in clinical practice

Type of SA	Residents (*n* = 73)	Assistant Psychologists (*n* = 121)
Self-report questionnaires (e.g., BDI)	74.0%	78.5%
Structured interviews (e.g., DIPS)	53.4%	49.6%
Semi-structured interviews (e.g., SCID)	53.4%	59.5%
Standardized observational assessments (e.g., ADOS)	24.7%	34.7%
Cognitive tests (e.g., WISC)	49.3%	81.0%
Mandatory questionnaires (e.g., HoNOSCA)	45.2%	47.9%
None of the above	2.7%	2.5%

*ADOS* Autism Diagnostic Observation Schedule, *BDI* Becks Depression Inventory, *DIPS* Diagnostisches Interview bei psychischen Störungen = Structured Clinical Interviews for Mental Disorders Across the Life Span, *HoNOSCA* Health of the Nation Outcomes Scales for Children and Adolescents, *SCID* Structured Clinical Interview for DSM-5, *WISC* Wechsler Intelligence Scale for Children.

When comparing RES and AP, there were no differences in the frequency of using SA (t_192_ = 1.3, *p* = 0.20, Fig. 1A), personally administering SA in clinical practice (t_192_ = 1.7, *p* = 0.090), and using the results of SA collected by others (t_192_ = 1.4, *p* = 0.16).

### Personal interest in standardized assessments

**Fig. 2:**
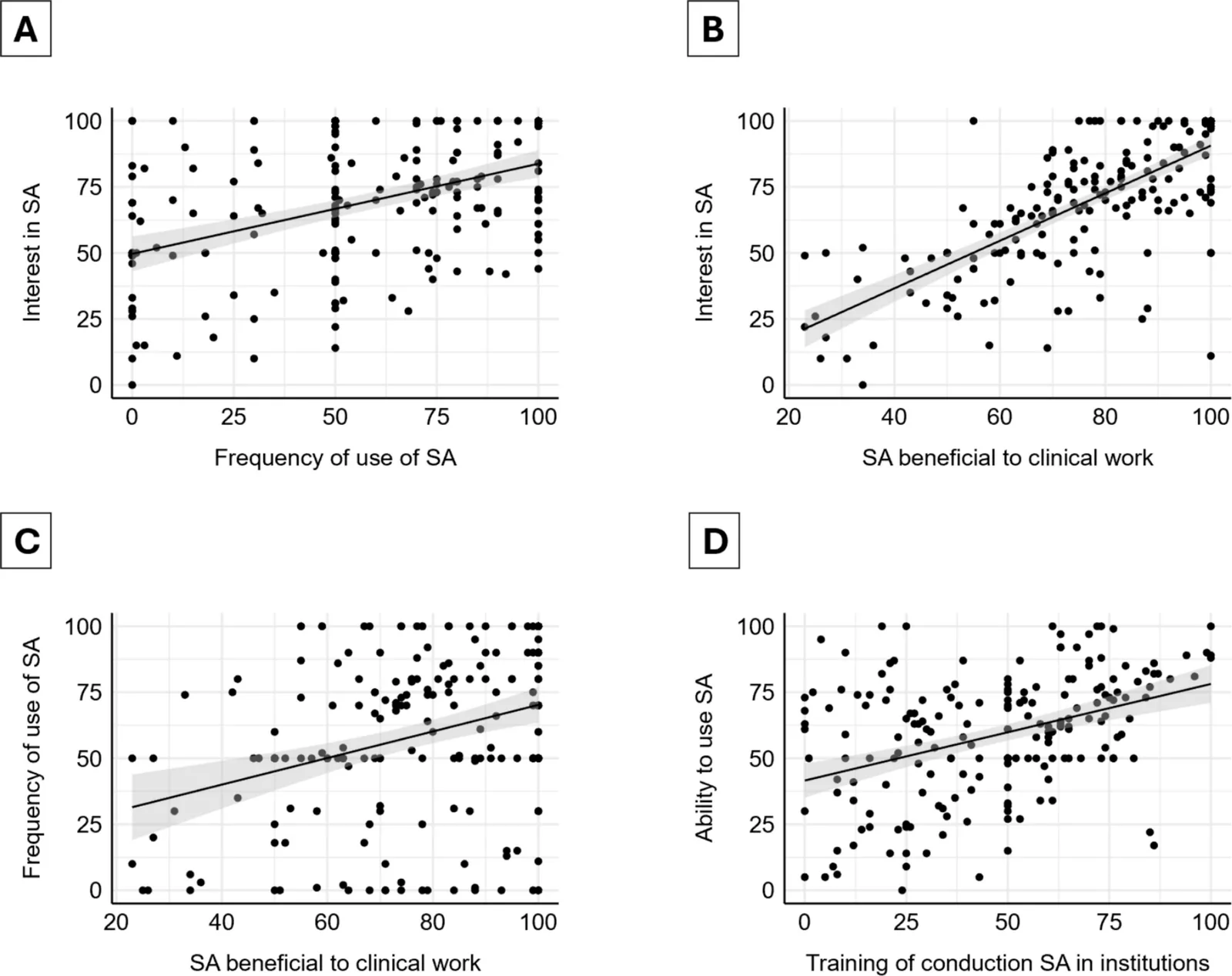
Correlation plots displaying associations between different variables included in the survey (**A**-**D**). Data from residents and assistant psychologists were combined, given there were no significant differences between the two groups. The 95% confidence interval is depicted as a shaded area around the regression line. SA = standardized assessments

Trainees reported being interested in SA (RES: 67.9/100 [SD 24.4, range 10—100], AP: 70.6/100 [SD 25.3, range 0—100], VAS from “not at all” (0) to “very much” (100), see Fig. 1B). Interest did not differ by type of psychotherapeutic training (F_4,756_ = 2.0, *p* = 0.093), institution (university clinic or not; F_1,189_ = 2.7, *p* = 0.10), scientific degree (Dr. med./PhD or none; F_1,189_ = 0.0, *p* = 0.94), or official language at the workplace (F_3,570_ = 0.80, *p* = 0.494). Interestingly, interest in SA was positively correlated with frequency of use, meaning interest increased with the frequency of SA use (τ = 0.31, *p* < 0.001, see Fig. 2A). No correlations were found between interest in SA and feeling involved in research (τ = −0.09, *p* = 0.13), time spent doing research at work (τ = −0.03, *p* = 0.65), or progression of training (τ = −0.04, *p* = 0.41).

When comparing the groups of RES and AP, there was no difference in interest in SA (t_192_ = 0.7, *p* = 0.46, see Fig. 1B). Among RES, but not AP, interest tended to be increased in institutions where ST was reported to be the main psychotherapeutic approach compared to PP (Profession x Main Psychotherapeutic Approach at Institution (MPAI): F_4,756_ = 2.8, *p* = 0.041, MPAI for RES: F_4,68_ = 2.7, *p* = 0.054; MPAI for AP: F_4,116_ = 0.7, *p* = 0.58; tendency of ST > PP in RES: t_29_ = 2.7, *p* = 0.058).

Trainees reported that they would use SA more often, if they were available, and they knew how to use and interpret them (RES: 75.8/100 [SD 22.5, range 9—100], AP: 74.5/100 [SD 22.9, range 0—100], VAS from “never” (0) to “very often” (100)). Trainees believe that SA are beneficial to clinical work (RES: 76.3/100 [SD 21.0, range 25—100], AP: 76.9/100 [SD 19.6, range 23—100], VAS from “not at all” (0) to “very helpful” (100), see Fig. 1C). Rating of clinical benefits of SA differed by psychotherapeutic training (F_4,756_ = 4.2, *p* = 0.003). Participants in CBT and CT training were more likely to report intention to use SA than participants in PP training (CBT vs. PP: t_83_ = 3.1, *p* = 0.027; CT vs. PP: t_94_ = 3.4, *p* = 0.010). The perceived clinical benefit of SA did not differ between the reported dominant psychotherapeutic approach at the workplace (F_4,756_ = 1.0, *p* = 0.39), or institution type (university hospital or not; F_1,189_ = 2.1, *p* = 0.15). There were highly positive correlations between perceived clinical benefit of SA and interest in SA (τ = 0.58, *p* < 0.001, see Fig. 2B), SA use frequency (τ = 0.20, *p* < 0.001, see Fig. 2C), and ability to use SA (τ = 0.20, *p* < 0.001). Top reasons for using SA in clinical practice were to support objectivity (RES: 75.3%, AP: 75.2%), to structure interviews (RES: 72.6%, AP: 65.3%), and to address sensitive topics with patients (RES: 58.9%, AP: 74.4%). Only a minority reported that SA save time (RES: 43.8%, AP: 30.6%) and very few reported using SA for research purposes (RES: 13.7%, AP: 6.6%). Reported risks and difficulties included a lack of time (RES: 75.3%, AP: 75.2%), the idea that SA are not useful (RES: 39.7%, AP: 36.4%), a lack of supervision (RES: 27.4%, AP: 16.5%) and resources (RES: 27.4%, AP: 22.3%), as well as the concern that SA might interfere with therapy (RES: 23.3%, AP: 28.1%). Other commonly noted drawbacks of SA not included in the specified answers, but mentioned in the free-text answers are the risk of undervaluation of the patients’ clinical impression and unprecise and misleading results (see supplementary material for further details) Finally, the lack of translation of SA into the patient’s language was a frequent barrier to their use (RES: 38.2/100 [SD 28.1, range 0—100], AP: 39.6/100 [SD 25.6, range 0—100], VAS from “never” (0) to “all the time” (100)).

There were no differences between RES and AP in stating that they would use SA more often if these were available, and they knew how to use and interpret them (t_192_ = 0.4, *p* = 0.68) and in their beliefs of clinical usefulness of SA (t_192_ = 0.2, *p* = 0.84).

### Knowledge and training in standardized assessments

Trainees rated their ability to use SA as moderate (RES: 49.7/100 [SD 25.1, range 0—100], AP: 64.1/100 [SD 20.8, range 6—100], VAS from “weak” (0) to “strong” (100), see Fig. 1D). Self-rating differed by psychotherapeutic training (F_4,756_ = 4.0, *p* = 0.004). Trainees with CBT training rated themselves higher than those in PP training (t_83_ = 3.16, *p* = 0.020). There were no differences between subjects with and without a scientific degree (Dr. med., PhD) (F_1,189_ = 0.7, *p* = 0.41). Importantly, the ability to use SA was positively correlated with institution training opportunities for conducting (τ = 0.29, *p* < 0.001, see Fig. 2D), scoring (τ = 0.25, *p* < 0.001), and interpreting (τ = 0.25, *p* < 0.001) SA. Interestingly, the ability to use SA was not correlated with the progression of participants’ training (τ = 0.04, *p* = 0.39). Training opportunities provided by institutions for conducting (RES: 42.8/100 [SD 26.1, range 0—100], AP: 49.2/100 [SD 25.0, range 0—100]), scoring (RES: 46.9/100 [SD 26.5, range 0—100], AP: 39.5/100 [SD 24.9, range 0—100]) and interpreting SA (RES: 40.5/100 [SD 25.4, range 0—100], AP: 46.1/100 [SD 25.2, range 0—100]) were rated rather low (all VAS from “non” to “extensive”). A vast majority of trainees reported that they would be interested in using SA, if resources and training opportunities were offered at their workplace (RES: 82.0/100 [SD 21.4, range 0—100], AP: 79.3/100 [SD 22.9, range 3—100], VAS from “not at all” to “yes for sure”).

When comparing RES and AP, the ability to use SA was rated higher among AP than RES (t_192_ = 4.3, *p* < 0.001, see Fig. 1D). Individual group comparisons revealed that the ability to use SA was higher among AP, but not among RES, working at a university hospital (Profession x Institution: F_1,189_ = 4.5, *p* = 0.034; Institution for AP: F_1,189_ = 7.0, *p* = 0.009, Institution for RES: F_1,189_ = 0.5, *p* = 0.48). There were no differences between RES and AP interest in SA training at the workplace (t_192_ = 0.8, *p* = 0.41).

## Discussion

Child and adolescent mental health is a key health priority in Europe and across the world. An increasing mental health burden among young people is merging with a growing shortage of mental health professionals, particularly child and adolescent psychiatrists. To explore a putative solution to strengthen CAP training as well as treatment quality and efficiency, we characterized a sample of CAP trainees (residents and assistant psychologists) in Switzerland and evaluated their SA use, skills, and views using an online survey. Results demonstrate that CAP trainees are interested in SA, use them frequently, and believe in their usefulness for clinical work. Our findings highlight the role and importance of SA in CAP and suggest that CAP programs should increase their SA resources, as well as clinical training and supervision opportunities.

Given the questionnaire was completed by about 25% of the residents and a substantial number of assistant psychologists in Switzerland, our survey also provides relevant information pertaining national CAP trainee demographics. As expected, trainees were mostly female. Close to 40% of the trainees reported having no research experience and only 30% of the residents completed a doctoral thesis (Dr. med.). The doctoral thesis completion rate across medical fields in Switzerland is unknown, but it is over double as high (63%) in Germany, a country with similar academic traditions [34]. While nearly all the assistant psychologists (95%) reported graduating in Switzerland, only a minority of the residents indicated obtaining their medical degree in Switzerland (37%), suggesting that the majority of them are foreign nationals. This finding highlights Switzerland’s significant dependence of foreign-trained CAP residents.

Overall, no substantial differences were found between the responses of residents and assistant psychologists pertaining interest, experience, and application of SA. Results indicate that both trainee groups are interested in SA, independent of their psychotherapy training background, and believe in the clinical usefulness of SA. Similar results pertaining positive attitudes of CAP clinicians towards SA were found in a study conducted in Sweden [17]. Our findings showed that although interest in SA did not differ between psychotherapy training backgrounds, frequency of use, ability to administer, and perception of clinical usefulness were significantly increased among participants with training in CBT compared to PP (a treatment approach where SA traditionally play a subordinate role). This suggests that SA use, skills, and attitudes may change through SA exposure.

Trainees rated their ability to perform SA moderately and expressed high levels of interest in free training. Importantly, participants’ self-rated ability to administer SA correlated positively with SA training opportunities in their respective institutions, indicating that training is effective. Reported SA use frequency was positively associated with SA interest. Moreover, trainees’ evaluation of the clinical usefulness of SA was correlated with frequency of use as well as interest in and ability to administer SA. Thus, SA training opportunities may have the potential to increase SA use, and in turn enhance SA interest and skills, as well as perceptions of clinical usefulness (note the caveat that these correlations do not prove causation). Importantly, regular SA application and interpretation training is likely critical to competency development and skill refinement, and feedback systems, reminders, as well as regular supervision practices may be necessary to achieve sustained SA use [30].

Trainees reported only receiving moderate support from their institutions in terms of training to conduct, score, and interpret SA, even though they are often responsible for nationally mandatory quality SA (i.e., the Health of the Nation Outcome Scales for children and Adolescents, HoNOSCA) during their training [35, 36]. This finding and the fact that CAP residents have ranked their training in evidence-based medicine to be the weakest of the 8 dimensions assessed by the Swiss Institute for Continuing Medical Education and Training (SIWF) [32], underscores the importance of teaching and promoting evidence-based practice (including the use of SA) in CAP. Trainees may be particularly motivated to implement SA via evidence-based practices like MBC due to direct feedback and interaction with the patient [28, 37, 38].

Given SA are efficient and cost-effective means to enhance patient care [26] and specialist training [39], we suggest that supporting their use may be a strategy to strengthen CAP moving forward. Based on our findings that trainees view SA positively and increased SA exposure is associated with positive attitudes towards SA, we propose firmly anchoring SA in specialist training programs as early as possible. SA accessibility and implementation can be facilitated by digital assessment tools [40, 41], and the best SA results and sustained practice are likely to be achieved through training and consultation sessions as well as long-term support systems (e.g., supervision, feedback and reminders systems) [30]. Broad, commonly used instruments such as the Mini International Neuropsychiatric Interview for Children and Adolescents (MINI-KID) [18] or the Diagnostisches Interview bei Psychischen Störungen (DIPS, Structured Clinical Interviews for Mental Disorders Across the Life Span) [42] can be helpful tools in clinical training contexts and also provide quality control of clinical judgement. A next step could be to develop state-of-the-art SA training programs for CAP trainees and assessing treatment results via trainee and patient feedback as well as standardized treatment quality assessments.

### Limitations of SA

There are also general limitations of SA, such as the risk of losing interest in the individual, not being able to capture individuals in their full complexity, cultural bias (due to validation only in specific populations), limited validity in complex cases, and significantly reduced clinical interaction. Practical barriers include necessary training and supervision, lack of translation and financial costs. Other assessment types, such as unstructured clinical interviews, unstandardized behavioral observation, or ecological momentary assessments may address some of these shortcomings. However, they include other limitations, as lack of objectivity and comparability, lower reliability, greater time investment, or low compliance. In summary, we are confident that advantages of SA mentioned above outweigh their limitations. Moreover, in line with the diagnostic gold standard, which integrates a combination of clinical evaluation and SA [43], different assessment modalities are not mutually exclusive and should therefore be combined to complement each other.

### Strengths and limitations

A strength of this study is that it is the first to characterize a large sample of CAP trainees in Switzerland and evaluate trainees SA use as well as their corresponding skills and attitudes. Limitations include that the study did not evaluate if SA use is associated with improved clinical treatment outcomes, better training quality, or residents’ training satisfaction. Participation rate was satisfactory, but may not be a comprehensively representative sample of CAP residents and assistant psychologists. The reported correlations, while revealing associations, do not prove causation and should be evaluated in further studies. Additionally, the study did not assess reasons for Switzerland’s shortage of child and adolescent psychiatrists, suggest explicit measures to increase the workforce, or provide specific strategies pertaining to how SA could be used to meet increasing treatment demands. These highly relevant and pressing challenges should be addressed by future studies, political decision makers, and society as a whole.

## Conclusions

This study aimed to characterize a sample of CAP trainees in Switzerland and to assess trainees’ use and attitudes towards SA. Based on the results and given the significant and increasing demand for CAP services, we propose that investing in trainees’ training programs as well as teaching and promoting SA on a low-threshold basis will be essential to ensuring effective and efficient evidence-based CAP services moving forward.

## Supplementary Information

Below is the link to the electronic supplementary material.

Supplementary file1 (PDF 334 KB)

## Data Availability

Data supporting the findings of this study are included in the article. Inquiries pertaining data availability can be directed to the corresponding author.

## Abbreviations

ADOS: Autism Diagnostic Observation Schedule
AP: Assistant psychologists
BDI: Becks Depression Inventory
CAP: Child and adolescent psychiatry
CBT: Cognitive behavioral therapy
CT: Combined therapy
DIPS: Diagnostisches Interview bei psychischen Störungen = Structured Clinical Interviews for Mental Disorders Across the Life Span
HoNOSCA: Health of the Nation Outcomes Scales for Children and Adolescents
MINI-KID: Mini International Neuropsychiatric Interview for Children and Adolescents
MBC: Measurement-based care
PP: Psychodynamic psychotherapy
RES: Residents
SA: Standardized assessments
SCID: Structured Clinical Interview for DSM-5
ST: Systemic therapy
UT: Unspecified therapy
VAS: Visual analogue scale
WISC: Wechsler Intelligence Scale for Children

## Declarations

### Author information

Matthias Liebrand and Maya Cosentino contributed equally as first authors. Paul Klauser and Jochen Kindler contributed equally as last authors.
