# Supplementary material for: Experience with standardized assessments in child and adolescent psychiatry: Findings from a national trainee survey in Switzerland
Source: Eur Child Adolesc Psychiatry. 2026 Jun 3;35(9):2991–3001. doi: 10.1007/s00787-026-03052-0 (PMC13695670; doi:10.1007/s00787-026-03052-0)
Supplement: Supplementary file 1 [file 787_2026_3052_MOESM1_ESM.pdf]

Supplementary Material

For the article ***Experience with standardized assessments in child and adolescent psychiatry: Findings from a national trainee survey in Switzerland*** in *European European Child & Adolescent Psychiatry*

The authors developed these materials to provide additional information and to keep the main manuscript from becoming needlessly long.

Supplemental Results

Below, the free text answers are detailed concerning limitations and risks of SA

- SA could lead to the clinical impression of the patient being overlooked (9x)
- Unprecise results or results could be misinterpreted (8x)
- Language Barrier (2x)
- SA do not do justice to the individual (2x)
- Lack of adaptation to cultural differences
- Less personal interaction with patient (to build psychotherapeutic realationship)
- SA neglect the personal expertise of the clinician
- Relational components could get lost
- SA's are time consuming
- The knowledge for interpretation of SAmight be missing,
- The patient doesn't trust SA
- SA are not applicable in small children

Contacted Institutions in Switzerland

**Location**	**Official name of CAP service**
Basel	Universitäre Psychiatrische Kliniken (UPK) Basel, Kinder- und Jugendpsychiatrische Klinik
Basel	Universitäre Psychiatrische Kliniken (UPK) Basel, Jugendforensik, Forensisch-Psychiatrische Klinik
Bellinzona	Organizzazione sociopsic. Cantonale, Servizio Medico Psicologico Sopraceneri, Sede Bellinzona e Locarno
Bern	Universitäre Psychiatrische Dienste Bern (UPD) Kinder- und Jugendpsychiatrie und Psychotherapie
Bern	Universitäre Psychiatrische Dienste Bern (UPD) Kinder- und Jugendpsychiatrie und Psychotherapie, Kinder- und Jugendforensik
Brig	Spitalzentrum Oberwallis, Kinder- und Jugendpsychiatrischer Dienst, PZO, Psychiatrie Zentrum Oberwallis

Chur	Kinder- und Jugendpsychiatrie Graubünden, Psychiatrische Dienste Graubünden (PDGR)
Delèmont	Centre médico-psychologique (CMP), Enfants et Adolescents (CMPEA)
Fribourg	Secteur de psychiatrie et de psychothérapie pour enfants et adolescents (SPPEA), Réseau Fribourgeois de Santé Mentale (RFSM)
Genf	Office médico-pédagogique
Genf	Institutions universitaires de psychiatrie, SPEA - Service de psychiatrie de l'enfant et de l'adolescent
Genf	Fondation Pôle Autisme
Lachen	Kinder- und Jugendpsychiatrie Uri, Schwyz und Zug
Lausanne	Centre hospitalier universitaire vaudois (CHUV), Service universitaire de psychiatrie de l'enfant et de l'adolescent (SUPEA)
Liestal	Kinder- und Jugendpsychiatrie, Psychiatrie Baselland
Littenheid	Clienia Littenheid AG, Zentrum für Kinder- und Jugendpsychiatrie und -psychotherapie
Locarno	Centro di perfezionamento in psichiatria et psicoterapia dell'infanzia e dell'adolescenza Ticino
Lugano	Servizio Medico Psicologico di Sottoceneri
Luzern	Kinder- und Jugendpsychiatrischer Dienst Luzerner Psychiatrie
Neuchâtel	Centre neuchâtelois de psychiatrie, Departement de psychiatrie de l'enfant et de l'adolescent
Schaffhausen	Kinder- und Jugendpsychiatrischer Dienst des Kantons Schaffhausen
Sierre	Hôpital du Valais, Psychiatrie enfant et adolescent, Hôpital de Sierre
Solothurn	Kinder- und Jugendpsychiatrie, Psychiatrische Dienste der Solothurner Spitäler AG
St. Gallen	Kinder- und Jugendpsychiatrische Dienste St. Gallen
Vevey	Fondation de Nant, Secteur psych. de l'Est vaudois, Service de psychiatrie et psychothérapie d'enfants et d'adolescents
Weinfelden	Kinder- und Jugendpsychiatrischer Dienst, Psychiatrische Dienste Thurgau, Psychiatriezentrum Weinfelden
Windisch	Psychiatrische Dienste Aargau AG (PDAG), Kinder- und Jugendpsychiatrischer Dienst, Klinik für Kinder- und Jugendpsychiatrie
Winterthur	Kantonsspital Winterthur, Sozialpädiatrisches Zentrum (SPZ)
Winterthur	Integrierte Psychiatrie Winterthur (IPW), Kinder- und Jugendpsychiatrie und -psychotherapie
Zürich	Psychiatrische Universitätsklinik Zürich (PUK), Kinder- und Jugendpsychiatrie und -psychotherapie
Zürich Forensik	Psychiatrische Universitätsklinik Zürich, Zentrum für Kinder- und Jugendforensik, Klinik für Forensische Psychiatrie
Zürich	Zentrum für Angst und Depressionsbehandlung Zürich
Zürich Kinderspital	Kinderspital Zürich, Abteilung Psychiatrie und Psychosomatik, Universitäts-Kinderklinik

Reference: https://www.siwf-register.ch/ (institution names partially corrected)

The Survey

This is the original survey as it was distributed among residents and assistant psychologists.

This survey will take about 15 minutes to complete. The purpose of this survey is to learn about the attitudes, interests and use of standardized assessments as well as better understand current research activity among residents and psychologists working in child and adolescent psychiatry in Switzerland. We hope the results will provide insight into how residents and psychologists use and perceive standardized assessments as well as reveal their current and past research activity.

Your participation is voluntary and by completing the following survey, you are giving your consent to participate and for usage of given data for research purposes. All of your responses will be completely anonymous, such that your email will not be linked to your survey and no conclusions about individuals can and will be made.

Thank you very much for your participation!

If you are interested in discussing this research please contact Matthias Liebrand or Maya Cosentino.

PERSONAL INFORMATION

Please leave us a specific code such that we can link your answers with those in a possible second wave of the survey. The code consists of the first two letters of your mother's first name, then the first two letters of your father's first name and then the year of your birth in four numbers, all lowercase (e.g., Judy, John and 1990: "jujo1990").

How old are you?
→ A number between 20 and 65 had to be selected

What is your gender?

- Female
- Male
- Divers

How many children do you have?
→ A number between 0 and 10 had to be selected

Are you a psychologist or resident (doctor)?

- Psychologist in training
- Certified Psychologist
- Resident (Physician in training)
- Physician with specialization
- Other

Please specify your profession.
→ This is a conditional question that only showed up if “Other” was selected in the previous question
→ The question had to be answered in a free text field

Do you work in child and adolescent psychiatry, in adult psychiatry or in another department?

- Child and adolescent psychiatry
- Adult psychiatry
- Other

Please specify the type of your department.
→ This is a conditional question that only showed up if "Other" was selected in the previous question
→ The question had to be answered in a free text field

What is your current contract-based workload?
→ VAS from "0%" to "100%," "50%" was displayed in the middle. The selected number could be seen.

Do you have a Dr. med., PhD or other research experience? (select all that apply)
→ It was possible to select multiple answers

- Dr. med.
- PhD
- Other research experience (including research during BA/MA/medical studies)
- None of the above

What year did you receive your (most recent) 2023 medical/psychology degree?
→ A year between 1990 and 2023 had to be selected

In what country did you receive your (most recent) degree?
→ One of 195 counties had to be selected

How many months of clinical child and adolescent psychiatry work experience have you completed (in full-time equivalent)?
→ Had to be answered in a free text field

In which canton do you currently work?
→ One of the 26 cantons had to be selected

What type of care facility do you mainly work at the moment (i.e. your ward)? (select all that apply)
→ It was possible to select multiple answers

- Outpatient
- Inpatient
- Day care facility
- Other

Please specify the type of facility you are working in.
→ This is a conditional question that only showed up if "Other" was selected in the previous question
→ The question had to be answered in a free text field

What type of institution do you currently work at? (select all that apply)
→ It was possible to select multiple answers

- University Hospital
- Public Non-University Hospital

- Private Hospital
- Private Practice
- Combined public-private Hospital
- Other

Please specify the type of institution you are working in.
→ This is a conditional question that only showed up if "Other" was selected in the previous question
→ The question had to be answered in a free text field

Is research being conducted at your institution?
- Yes
- No
- I don't know

Are you currently involved in research activities at your institution?
→ VAS from "Not at all" to "Very much"

How many hours do you do research per week?
→ VAS from 0 to 50

STANDARDIZED ASSESSMENTS

Do you personally use standardized assessments (not including the HoNOSCA or the GAF) collected by yourself or others in your clinical practice?
→ VAS from "Never" to "Always". "Sometimes" was displayed in the middle.

How often do you use standardized assessments (not including the HoNOSCA or the GAF) collected by yourself in your clincal practice?
→ VAS from "Never" to "Always". "Sometimes" was displayed in the middle..

How often do you use information from standardized assessments (not including the HoNOSCA or the GAF) collected by others in your clinical practice?
→ VAS from "Never" to "Always". "Sometimes" was displayed in the middle.

What are standardized assessments used for in your institution? (select all that apply)
→ It was possible to select multiple answers
- Diagnosis
- Screening
- Psychotherapy treatment monitoring / outcomes
- Pharmacological treatment monitoring / outcomes
- General outcome
- Research
- They are not used

Which of the following standardized assessments do you Self-report questionnaires (e.g., BDI) personally use in your clinical practice? (select all that apply)
→ It was possible to select multiple answers
- Self-report questionnaires (e.g., BDI)
- Structured interviews (pre-determined set of questions, e.g., DIPS)
- Semi-structured interviews (also open questions, e.g., SCID)

- Standardized observational tasks (e.g., ADOS)
- Cognitive tests (e.g., WISC)
- Mandatory questionnaires (e.g., HoNOSCA)
- None of the above
- I don't know

Which disorders do you or your colleagues assess using standardized assessments in your institution? (select all that apply)
→ It was possible to select multiple answers

- AD(H)D
- Addictive behaviors
- Anxiety
- Autism spectrum disorder
- Bipolar disorder
- Childhood emotional/behavioral disorders
- Depression
- Dissociative disorders
- Eating disorders
- Gender dysphoria
- Intelligence/cognitive abilities
- Obsessive compulsive disorders
- Personality disorders
- Psychosomatic disorders
- Schizophrenia/psychosis
- Trauma
- Other
- None

Please specify the assessment.
→ This is a conditional question that only showed up if "Other" was selected in the previous question
→ The question had to be answered in a free text field

When are standardized assessments conducted/used in or shortly after admission your institution? (select all that apply)
→ It was possible to select multiple answers

- At or shortly after admission
- During treatment
- At discharge
- Not at all

PERSONAL INTEREST IN STANDARDIZED ASSESSMENTS

How interested are you in using standardized assessments?
→ VAS from "not at all" to "very much"

If standardized assessments (not including the HoNOSCA or the GAF) were available and you knew how to use and interpret them, would you use them?
→ VAS from "never" to "very often"

Do you believe standardized assessments are beneficial to clinical work (e.g., diagnosis, progress, satisfaction with treatment, adherence)?
→ VAS from "not helpful at all" to "very helpful"

What reasons do you have for using standardized assessments at work? (select all that apply)
→ It was possible to select multiple answers

- Standardized assessments can help me address sensitive topics with patients
- Standardized assessments can help me structure interviews
- Standardized assessments can save me time
- The data collected with standardized assessments support objectivity
- I use data collected with standardized assessments for research purposes
- Other reasons
- None of the above

Please specify the other reason(s) for using standardized assessments.
→ This is a conditional question that only showed up if "Other reasons" was selected in the previous question
→ The question had to be answered in a free text field

If you do not use standardized assessments in your clinical practice, what might be the reason? (select all that apply)
→ It was possible to select multiple answers

- I don't know of any assessment tools that would be useful
- I do not have or know how to access to any standardized assessments
- Nobody I work with uses standardized assessments
- I never learned how to use and/or interpret standardized assessments
- I do not have enough time to use standardized assessments
- I don't see the clinical relevance of using standardized assessments
- Standardized assessments do not exists in the languages I would need
- Standardized assessments are only used by researchers
- Standardized assessments are only used by psychologists (only applies to residents)
- Other reasons
- None of the above

Please specify the reason(s) to not use standardized assessments in your clinical practice.
→ This is a conditional question that only showed up if "Other reasons" was selected in the previous question
→ The question had to be answered in a free text field

What are possible risks or difficulties for you associated with standardized assessments at work? (select all that apply)
→ It was possible to select multiple answers

- Standardized assessments could interfere with my therapeutic relationship
- I don't have enough time to use and interpret standardized assessments
- There is no one at my institution to supervise my use/interpretation of standardized assessments
- There are insufficient resources/knowledge available at work to use standardized assessments
- I am unsure which standardized assessments could be useful
- Other risks/difficulties
- None of the above

Please specify the other risks/difficulties.
→ This is a conditional question that only showed up if "Other risks/difficulties" was selected in the previous question
→ The question had to be answered in a free text field

KNOWLEDGE AND TRAINING IN STANDARDIZED ASSESSMENTS

How would you rate your ability to use standardized assessments?
→ VAS from "weak" to "strong"

Does your institution provide or facilitate training in conducting standardized assessments?
→ VAS from "none" to "extensive"

Does your institution provide or facilitate training in scoring of standardized assessments?
→ VAS from "none" to "extensive"

Does your institution provide or facilitate training in interpreting standardized assessments?
→ VAS from "none" to "extensive"

Where have you learned about standardized assessments? (select all that apply)
→ It was possible to select multiple answers

- Supervisors at work
- Other work colleagues
- Clinic/hospital management
- Supervision (not at work)
- Past work experience
- Psychotherapy training program
- General residents training
- On my own
- Research activity
- Other
- I have no knowledge about standardized assessments

Please specify where you have learned about standardized assessments.
→ This is a conditional question that only showed up if "Other" was selected in the previous question
→ The question had to be answered in a free text field

If free training on the use of standardized assessments is/was offered at your workplace do/would you make use of it?
→ VAS from "not at all" to "yes for sure"

PERSONAL AND INSTITUTIONAL BACKGROUND

Which type of psychotherapy training are you attending or have you already completed?

- Psychodynamic psychotherapy
- Cognitive Behavioral Therapy (CBT)
- Systemic Therapy
- A combination (e.g., CBT and Systemic Therapy)
- Other

Please specify the combination of psychotherapeutic training you attend/completed.
→ This is a conditional question that only showed up if "A combination" was selected in the previous question
→ The question had to be answered in a free text field

Please specify what type of training you are attending or have completed.
→ This is a conditional question that only showed up if "Other" was selected in the previous question
→ The question had to be answered in a free text field

What is the predominant psychotherapeutic approach at your workplace?

- Psychodynamic psychotherapy
- Cognitive Behavioral Therapy (CBT)
- Systemic Therapy Other
- None in particular

Please specify the predominant psychotherapeutic approach at your clinic.
→ This is a conditional question that only showed up if "Other" was selected in the previous question
→ The question had to be answered in a free text field

What is/are the official language(s) at your workplace? (multiple answers possible)
→ It was possible to select multiple answers

- German
- French
- Italian

Has the lack of a standardized assessment's translation been a barrier to its use?
→ VAS from "never" to "all the time"

What is the ratio of psychologists to physicians at only your workplace (e.g., ward or outpatient unit)?
→ VAS from "only physicians" to "only psychologists"

COMMENTS

Do you have any comments regarding the survey? Please let us know!
→ The question could to be answered in a free text field
